# A Novel Light Field Image Compression Method Using EPI Restoration Neural Network

**DOI:** 10.1155/2022/8324438

**Published:** 2022-06-13

**Authors:** Jinghuai Liu, Qian Zhang, Ang Shen, Ying Gao, Jiaqi Hou, Bin Wang, Tao Yan

**Affiliations:** ^1^College of Information, Mechanical and Electrical Engineering, Shanghai Normal University, Shanghai 200234, China; ^2^Distribution Grid Dispatching and Control Center, State Grid Qingdao Power Supply Company, Qingdao, Shandong 266001, China; ^3^School of Mechanical, Electrical & Information Engineering, Putian University, Putian, Fujian 351100, China

## Abstract

Different from traditional images, light field images record not only spatial information but also angle information. Due to the large volume of light field data brings great difficulties to storage and compression, light field compression technology has attracted much attention. The epipolar plane image (EPI) contains a lot of low rank information, which is suitable for recovering the complete EPI from a part of EPI. In this paper, a light field image coding framework based on EPI restoration neural network has been proposed. Compared with previous algorithms, the proposed algorithm further takes advantage of the inherent similarity in light field images, and the proposed framework has higher performance and robustness. Experimental results show that the proposed method has superior performance compared to the state-of-the-art both in quantitatively and qualitatively.

## 1. Introduction

**Figure 1:**
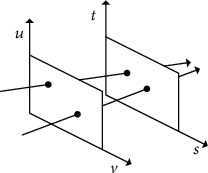
Four-dimensional light field model.

With the development of new technology, the three-dimensional world can be more abundant and immersive sampling. Light field imaging technology is one of the main ways to collect three-dimensional scene information. Objects and scenes in the real world can be represented by the light field, which is widely used in refocusing, view synthesis, depth estimation, three-dimensional reconstruction, and so on. The all-optical function *I* = *P*(*x*, *y*, *z*, *θ*, *ϕ*, *ω*, *t*) was proposed by Adelson and Bergen [1], which was used to describe the position, direction, time, and wavelength information of light in three-dimensional space. Nevertheless, it was difficult to record and process this kind of high-dimensional information. Levoy and Hanrahan [2] further simplified the full-light model and proposed a four-dimensional light field model *L*(*u*, *v*, *s*, *t*), where (*u*, *v*) was used to describe the angle information of the light field and (*s*, *t*) was used to describe the spatial information of each angle of the light field. The four-dimensional light field model is shown in Figure 1.

The light field images contain not only angular information of light distribution but also spatial information, which means that the light field contains a large amount of high-dimensional redundant data. Raw data can range from hundreds of megabytes to thousands of gigabytes depending on light field sampling and capture equipment. For example, the Lytro Illum (microlens array light field camera) can collect light from 225 directions, generating about 1.82 GB of light field data [3]. In order to store, transmit, and render light field images, a more efficient encoding scheme is required.

The mainstream light field image coding methods are closely related to video coding methods. For example, a subaperture image stream compression scheme is proposed in [4], which takes a subaperture image as a frame in a video and encodes it with standard video compression methods. Yet, only using video compression methods is not enough; it does not take advantage of the relationship between nonadjacent subaperture images. Monteiro et al. [5] optimized the intrablock prediction model of HEVC, not only using the translation prediction model but also adding the projection and bilinear transformation model. Jia et al. [6] combine deep learning algorithms with HEVC. Unsampled subaperture images are predicted by generation adversarial networks from sampled subaperture images. However, this deep learning-based framework relies heavily on the previous training data. Once the content of light field data changes, it will face a very time-consuming retraining process.

In this paper, we consider that there is a large amount of low-rank information in EPI and propose an efficient light field image compression method. Wu et al. [7] proposed an algorithm framework for recovering a complete EPI from a sampled EPI. We get inspiration from this and propose a framework of light field image coding algorithm. Therefore, we integrate this structure into the light field image coding framework. Firstly, the optical field subaperture image is sampled and encoded with VVC. Then, in the decoding stage, the sampled subaperture image is transformed into EPI form. Finally, a recovery algorithm module is used to restore a complete EPI. The main contributions of this paper are listed as follows:

- The paper presents a novel framework for the compression of light field images by using the relationship between subaperture images and EPI
- The EPI is sampled and then restored to facilitate the compression of the low-rank part
- The proposed compression scheme presents a generalized solution for light field images

The remainder of this paper is organized as follows: In Section 2, several relevant solutions about light field image compression are described.  Section 3 introduces the proposed light field image coding framework. In Section 4, the simulated experimental results and test conditions are presented. Ultimately, this paper is concluded in Section 5.

## 2. Related Work

Several coding schemes of light field images described in the literature all take advantage of the redundancy of light field images. These schemes depend on different image representation and coding techniques, and their basic methods can be divided into three categories: transform-based coding, pseudo video sequence coding, and prediction-based coding.

Some low-frequency signal coding schemes essentially rely on the use of the transform, such as Discrete Cosine Transform (DCT) [8] or Discrete Wavelet Transform (DWT) [9]. After several micro images are stacked together, 3D-DCT is used to remove the spatial redundancy between adjacent micro images. The resulting transformation coefficients are then quantified and entropy encoding. In [9], light field images are decomposed into view images, and 3D-DWT is performed on a group of view images. 2D-DWT is used to transform the low-frequency part, and then, arithmetic coding is carried out, while the other high-frequency coefficients are simply quantized and arithmetic coded. Since the light field image can be regarded as a four-dimensional form, the 4D-DWT is used to compress the light field images in [10]. In [11], a light field coding method based on 4D-DCT is proposed, which introduces four-dimensional quantization and block traversal. In [12], each subview image is regarded as the node of the graph, and the similarity between adjacent views is regarded as the edge of the graph. At the coding end, the optimal graph representation is obtained by minimizing the sparsity and connectivity of the graph. According to the graph representation, the reference viewpoint is selected, and the light field image is compressed by graph transform.

Recently, HEVC is used to process light field data in many solutions [13]. HEVC specializes in using internal prediction tools to eliminate redundancy in traditional two-dimensional images. Nevertheless, it does not carry out specific processing according to the characteristics of the light field image, so many researchers put forward corresponding improvement measures. The method based on pseudo video sequence was studied in [14], mainly changing the order of subaperture images to deal with the redundant interference between subaperture images. Various scanning sequences, such as rotation sequence [4], raster scanning sequence [14], horizontal zigzag, and U-shaped mixed scanning sequence [15], are studied in the process of pseudo video sequence generation. Liu et al. improved on HEVC [16]; the prediction unit (PU) is divided into three different categories, and different prediction models based on Gaussian process regression are used for each category. In [17], a new light field multiview coding prediction structure is designed, which extends the interview prediction to a bidirectional parallel structure, and analyzes the relationship between the prediction structure and the coding performance. Because of the high similarity between subaperture images, Helin et al. [18] proposed a method of lossless compression with correction for low-frequency images.

With the improvement of hardware performance, deep learning technology has developed rapidly, attracting more and more attention from researchers and has been applied in many fields. For example, Coherence Constrained Graph LSTM (CCG-LSTM) with Spatio-Temporal Context Coherence (STCC) and Global Context Coherence (GCC) are proposed to recognize group activities [19]. Skeleton-joint Coattention Recurrent Neural Networks are proposed to generate future motions based on the observed human motions [20]. Expansion-Squeeze-Excitation Fusion Networks (ESE-FN) are proposed to recognize elderly activity [21]. Chen et al. [22] proposed a prediction scheme based on HEVC, in which a group of the sparse subaperture images is encoded in a base layer. Other subaperture images in the decoder are reconstructed by using interpolation. The reconstructed image is then used to predict the entire light field image, and a prediction residual is transmitted. In [23], the author uses a convolutional neural network to predict all the views from the four corners, while the author in [24] uses a DIBR (depth-image-based rendering) method, by estimating the depth and using the depth-reference view to predict the other views. In [25], firstly, a set of selected subaperture images are taken as reference views and then encoded into video sequences by HEVC and transmitted to the decoder. Finally, the unselected image is reconstructed from the decoded subset of the selected image by using the low-frequency sparsity of the angular continuous Fourier domain.

Monteiro et al. [5] proposed a two-stage block high-order prediction model by using eight degrees of freedom geometric transformation. In [26], a minimum mean square error estimation method based on scalable kernel is proposed to accelerate the prediction process of light field image coding. Based on the inherent nonlocal spatial redundancy of low-frequency images, a coding method combined with local linear embedding is proposed in [27]. Monteiro et al. [28] proposed a method based on locally linear embedding and a compensation prediction method. In [24], a sparse predictor is used to predict multiple reference points. The disparity compensation wavelet coding technology is applied in [29]. Huang et al. [3] studied content consistency and structure consistency to achieve efficient light field compression at a low bit rate.

As mentioned above, according to the characteristics of light field images, the above methods combine 4D-DCT, linear approximation, convolutional neural network, and other methods with traditional video coding algorithms to achieve the purpose of compression of light field images. However, there are some problems with this approach. For example, the linear relationship between nonadjacent subaperture images is not properly handled, the coding complexity is high, and the data need to be trained in advance and time-consuming. In this paper, the relationship between subaperture images can be linked by EPI, which can be restored from a partial EPI. Therefore, we can compress the light field image by the characteristic of EPI.

## 3. Proposed Coding Solution

**Figure 2:**
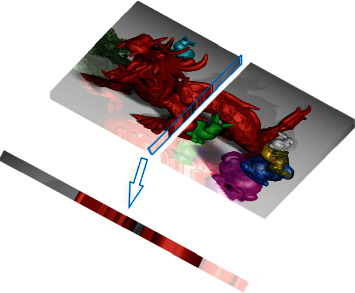
EPI generation method. Stacking a row of subaperture images, the cross section is the epipolar plane image.

As EPI contains both angle information and spatial information of light field, the light filed image compression method proposed in this paper focuses on processing of EPI. As shown in Figure 2, EPI consists of pixels of the same number of rows of each subaperture image. We consider sampling the EPI in coding and then recovering the original EPI in decoding, so as to reduce the amount of data transferred. Since the parallax between adjacent subaperture images of dense light field is less than 1 pixel, the sampling of EPI meets the Nyquist sampling rate, and the original EPI can be restored according to the all-optical function.

Due to the characteristics of light field image, it contains not only spatial domain information but also angular domain information. The light field EPI connects the spatial information of the light field with the angle information in its own way. VVC is optimized on the basis of HEVC and obtains the maximum coding gain as far as possible by improving every link of coding. VVC can save about 50% bit-rate compared with HEVC at the same quality. Therefore, this paper starts with EPI, builds an image coding network based on EPI enhancement, and combines it with the existing video coding standard VVC to obtain a more efficient compression coding scheme for light field images.

Wu et al. [7] proposed a light field image reconstruction network. Its main idea is to add blur before recovering sampled images using CNN and remove blur after upsampling, which can make reconstructed images have better quality than those without the process of “blur-restoration-deblurring.” Based on this framework, this paper constructs a more suitable frame for light field image coding. Due to different problems to be solved, Wu et al.'s problem is to reconstruct the light field with high angular resolution from the light field with low angular resolution, and they do not know the true value of the light field with high angular resolution to be reconstructed in advance. However, in this paper, the original high-resolution light field is sampled and reduced to the light field with low angular resolution. This process is then reconstructed into the original high-resolution light field, the values of which are known. Therefore, in this paper, two-dimensional kernel function is used to blur the original high-resolution EPI of the light field, instead of the one-dimensional function used after sampling. In this way, the blur can be carried out in two-dimensional as a whole, instead of in one-dimensional direction, so that the reconstructed light field image quality is higher.

**Figure 3:**
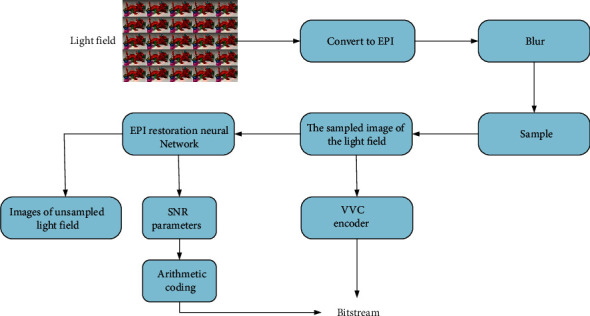
The framework of light field image coding. Firstly, the light field images are selected by the select module. The selected subaperture images are converted into EPIs through the rearrange module. Restore the EPIs through the restoration module. Finally, the selected subaperture image and the residual value are transmitted to the VVC encoder.

The whole workflow of the light field image compression coding framework proposed in this paper is shown in Figure 3. Firstly, the original light field image is transformed into EPI form; EPI is blurred by two-dimensional kernel function. Secondly, EPI is downsampled. Finally, VVC encoder is used to encode the selected sampled light field image, and EPI recovery network is used to recover the sampled light field image for the noncoded part.

**Figure 4:**
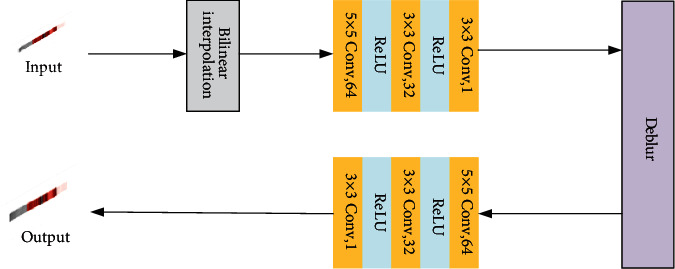
Network framework of recovery model.

The overall framework of the EPI recovery model is shown in Figure 4. The input is the EPI after blur processing and then sampling. First, the pixels to be restored are initialized by bilinear interpolation, and low-frequency details are recovered by three-layer convolutional network. Then, a deblur module is used to recover the high frequency detail features. Finally, the noise generated in the deblur process is removed through three-layer convolutional network, and the restored EPI is obtained.

Bilinear interpolation is used to initialize the pixels to be restored, which is convenient for subsequent convolutional neural network processing. The three-layer convolutional network is used to recover low frequency details of EPI. The first layer contains 64 convolution kernels with a size of 1 × 5 × 5 for feature extraction. The second layer contains 32 convolution kernels with a size of 64 × 3 × 3 for nonlinear mapping. The last layer contains a convolution kernel with a size of 32 × 3 × 3 for detail reconstruction. The activation function between the first and second layers is ReLU. Using ReLU is more efficient than other activation functions, does not have the problem of gradient disappearance, and prevents over fitting. The EPI is filled with zeros before each convolution to keep the input and output sizes the same. In the deblur module, Wiener filtering is used to deblur. Use arithmetic encoding SNR parameter. Finally, a three-layer convolutional network is used to deal with the noise generated in the process of deblur. The first layer contains 64 convolution kernels of size 1 × 5 × 5. The second layer contains 32 convolution kernels of size 64 × 3 × 3. The last layer contains a convolution kernel of size 32 × 3 × 3.

In order to ensure the high quality signal recovery and perceived quality of CNN, this paper adopts the mean square error loss function in the model. The formula of loss function is defined as follows:

(1) $$\begin{array}{c} L=\frac{1}{n}\overset{n}{\underset{i=1}{\sum }}{\left\|E^{i}-E^{\prime i}\right\|}^{2}, \end{array}$$

where *n* represents the number of trained EPI, *E* represents the original EPI, and *E*′ represents the EPI for detail recovery through convolutional neural network.

## 4. Experimental Results

In order to verify the performance of the proposed light field image coding framework, the coding efficiency of light field image is tested. A set of comparative experiments are designed to verify the performance of the proposed method. The light field image compression coding algorithm proposed in this paper is compared with the method proposed by Liu et al. [13], Chen et al. [22], and Jia et al. [6]. During the experiment, the standard deviation of Gaussian blur kernel was set as 0.83, and the selected subaperture image after blur was constructed into pseudo video sequence according to sinuous array. VVC coding software VTM-12.2 was used to compress and encode pseudo video sequence, and the coding method was set as “Random Access.” In the initialization parameter setting of convolutional neural network, the total number of iterations of training is set as, and the learning rate is set as 0.01. Each iteration, the learning rate attenuates to one-tenth of the original. The batch size is set to 64, and the momentum parameter is set to 0.9. The Gaussian distribution function with mean value 0 and standard deviation is used to initialize the convolution kernel of the convolutional neural network.

**Figure 5:**
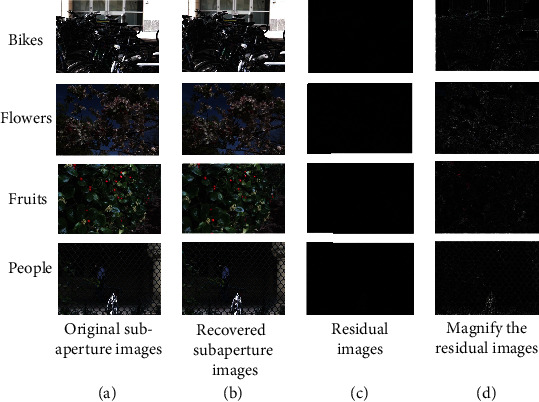
Experimental comparison of subaperture image restoration: (a) original subaperture image; (b) repaired subaperture image; (c) residual images; (d) magnify the residual images.

In this paper, “People,” “Bikes,” “Fruits,” and “Flowers” in Stanford light field data set are selected for the experiment. In order to facilitate the observation of experimental results, this paper presents the original image of one of the subaperture images in each light field before the experiment, the restored result image after the experiment, and the residual image of the two images. The experimental results are shown in Figure 5. The original subaperture image is on the far left, followed by the restored subaperture image. The residual image is obtained by subtracting the pixels of the original subaperture image and the restored subaperture image. It can be seen from the figure that most areas of the residual image are black, indicating that the residual value of the image is very small, and the difference between the restored subaperture image and the original subaperture image is very small; that is, the image restoration effect of the EPI restoration model is good. In order to facilitate the observation of the difference between the original subaperture image and the restored subaperture image, the pixel value of the residual image is enlarged to obtain the right-most residual enlarged image. It can be seen that the difference is mainly concentrated in the edge part of the object in the subaperture image.

Peak signal-to-noise ratio (PSNR) is the average value of all subaperture images, and its formula is defined as follows:

(2) $$\begin{array}{c} \text{PSNR}=\frac{1}{7\times 7}\overset{7}{\underset{u=1}{\sum }}\overset{7}{\underset{v=1}{\sum }}\text{P}\text{SNR}\left[u\right]\left[v\right]. \end{array}$$

The peak signal-to-noise ratio of each subaperture image is calculated as follows:

(3) $$\begin{array}{c} \text{PSNR}\left[u\right]\left[v\right]=10*\log_{10}\left[\frac{{\left(2^{n}-1\right)}^{2}}{\text{MSE}}\right],\\ \text{MSE}=\frac{1}{s\times t}\overset{s}{\underset{i}{\sum }}\overset{t}{\underset{j}{\sum }}{\left({\text{SAI}}_{r}\left[i\right]\left[j\right]-{\text{SAI}}_{o}\left[i\right]\left[j\right]\right)}^{2}, \end{array}$$

where *H* and *W* are the spatial resolution of each subaperture image, SAI_*r*_ and SAI_*o*_ represent reconstructed subaperture image and uncompressed subaperture image, respectively, and *n* indicates the bit depth.

**Figure 6:**
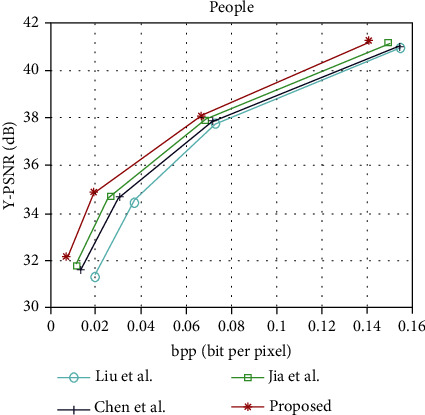
Rate-distortion curves of “People.”

**Figure 7:**
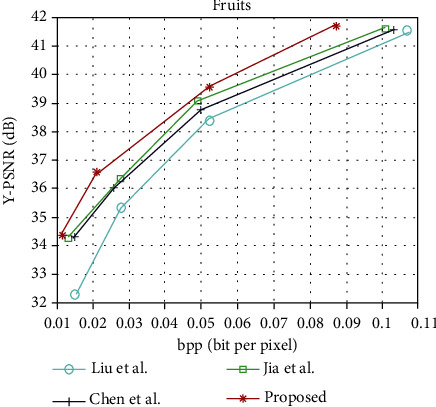
Rate-distortion curves of “Fruits.”

**Figure 8:**
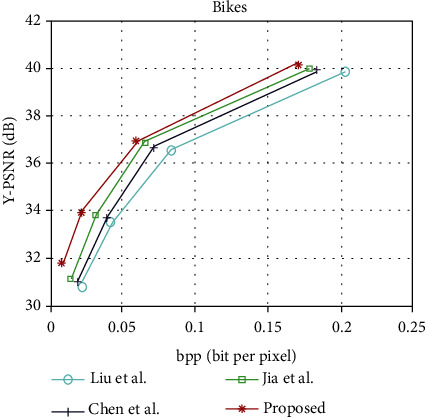
Rate-distortion curves of “Bikes.”

**Figure 9:**
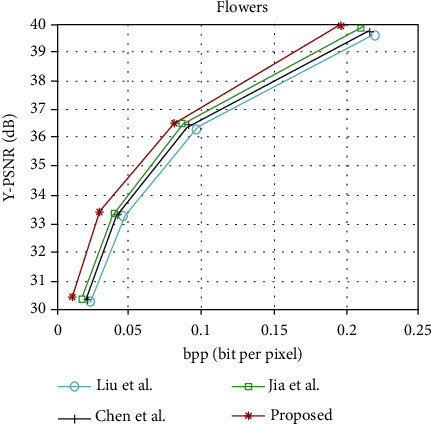
Rate-distortion curves of “Flowers.”

The light field image compression coding algorithm is proposed in this paper and the rate-distortion curves of Liu et al. [13], Chen et al. [22], and Jia et al. [6], under the light field data set “People,” “Fruits,” “Bikes,” and “Flowers.” The rate-distortion curves of “People,” “Fruits,” “Bikes,” and “Flowers” are shown in Figures 6–9, respectively. It can be seen from the figure that the performance of the light field image compression and coding algorithm proposed in this paper is superior to that proposed by Liu et al., Chen et al., and Jia et al.  Table 1 shows the rate-distortion performance in terms of BD-PSNR rate for each light field image. As can be seen from Table 1, the average BD-PSNR and average BD-BR of the light field image compression coding algorithm proposed in this paper are 2.02 dB and -39.47%, respectively. Compared with the light field image compression and coding algorithm proposed by Chen et al., the average BD-PSNR increases by 0.64 dB, and the average BD-BR decreases by 15.58%. Compared with the light field image compression algorithm proposed by Jia et al., the average BD-PSNR is improved by 1.08 dB, and the average BD-BR is reduced by 24.87%. Experimental results show that the proposed image compression coding algorithm can ensure the image quality while reducing the bit rate.

In order to prove the improvement of the proposed module over the method proposed by Wu et al., an ablation experiment was added in this paper. One group directly uses the network built by Wu et al. on the basis of VVC coding; the other group uses the network structure improved in this paper. We also conducted experiments on “People,” “Fruits,” “Bikes,” and “Flowers” light field data sets. The experimental results are shown in Table 2. The improved light field image coding performance is better than the method proposed by Wu et al.

## 5. Conclusion

In this paper, a new light field image coding algorithm framework is constructed. In order to ensure that the quality of the original light field EPI is recovered from the sampled light field EPI, the light field EPI is blurred first and then sampled. The sampled EPI was transformed into pseudo video sequence, which was encoded by the VVC reference software VTM-12.2. The unselected light field EPI is reconstructed by EPI recovery network model. In order to test the performance of the light field image compression, compared with the light field image compression and coding algorithm proposed by Liu et al., Chen et al., and Jia et al., the bit rate is relatively reduced, and the image quality is relatively higher, which confirms the superior performance of the light field image compression.

## Figures and Tables

**Table 1:** BD-PSNR (dB)/BD-BR (%): performance calculated with respect to the anchor method proposed by Liu et al.

Sequence	Chen et al. [22]		Jia et al. [6]		Proposed	
	BD-PSNR	BD-BR	BD-PSNR	BD-BR	BD-PSNR	BD-BR
Bikes	0.61	-14.50	1.26	-29.01	2.11	-47.35
Flowers	0.35	-8.13	0.62	-13.97	1.61	-31.22
Fruits	0.86	-25.39	1.10	-31.41	1.93	-38.41
People	0.75	-14.29	1.35	-25.10	2.44	-40.88
Average	0.64	-15.58	1.08	-24.87	2.02	-39.47

**Table 2:** BD-PSNR (dB)/BD-BR (%): performance calculated with respect to the anchor method proposed by Liu et al.

Sequence	Wu et al. [7]		Proposed	
	BD-PSNR	BD-BR	BD-PSNR	BD-BR
Bikes	0.73	-15.60	2.11	-47.35
Flowers	0.43	-10.67	1.61	-31.22
Fruits	0.94	-25.22	1.93	-38.41
People	0.87	-19.52	2.44	-40.88
Average	0.74	-17.75	2.02	-39.47

## Data Availability

The data used to support the findings of this study are available from the corresponding author upon request.
